# Behavioural interventions for weight management in pregnancy: A systematic review of quantitative and qualitative data

**DOI:** 10.1186/1471-2458-11-491

**Published:** 2011-06-22

**Authors:** Fiona Campbell, Maxine Johnson, Josie Messina, Louise Guillaume, Elizabeth Goyder

**Affiliations:** 1 School of Health and Related Research, University of Sheffield, 30 Regent Court, 30 Regent Street, Sheffield, S1 4DA, UK

## Abstract

**Background:**

There is a rising prevalence of excessive weight gain in pregnancy and an increasing number of pregnant women who are overweight or obese at the start of the pregnancy. Excessive weight gain during pregnancy is associated with adverse maternal and neonatal consequences and increases the risk of long-term obesity. Pregnancy therefore may be a key time to prevent excessive weight gain and improve the health of women and their unborn child. This systematic review sought to assess the effectiveness of behavioural interventions to prevent excessive weight gain in pregnancy and explore the factors that influence intervention effectiveness.

**Methods:**

We undertook a systematic review of quantitative and qualitative evidence. This included a meta-analysis of controlled trials of diet and physical activity interventions to prevent excessive weight gain during pregnancy and a thematic synthesis of qualitative studies that investigated the views of women on weight management during pregnancy. A thorough search of eleven electronic bibliographic databases, reference lists of included studies, relevant review articles and experts in the field were contacted to identify potentially relevant studies.

Two independent reviewers extracted data. RevMan software was used to perform the meta-analyses. Qualitative data was subject to thematic analysis. Both quantitative and qualitative data were aligned using a matrix framework.

**Results:**

Five controlled trials and eight qualitative studies were included. The overall pooled effect size found no significant difference in gestational weight gain amongst participants in the intervention group compared with the control group (mean difference -0.28 95% CI -0.64 to 0.09). The study designs, participants and interventions all varied markedly and there was significant heterogeneity within this comparison in the meta-analysis (I^2 ^67%). Subgroup and sensitivity analysis did not identify contextual elements that influenced the effectiveness of the intervention.

In a thematic analysis of the qualitative studies, three major themes emerged relating to women's views of weight management in pregnancy: pregnancy as a time of transition and change, conflicting and contradictory messages and a perceived lack of control. When the results of both quantitative and qualitative data were aligned it was clear that some of the barriers that women described in achieving healthy weight gain in pregnancy were not addressed by the interventions evaluated. This may have contributed to the limited effectiveness of the interventions.

**Conclusions:**

Despite intense and often tailored interventions there was no statistically significant effect on weight gain during pregnancy. Inadequate and often contradictory information regarding healthy weight management was reported by women in qualitative studies and this was addressed in the interventions but this in itself was insufficient to lead to reduced weight gain. Multiple types of interventions, including community based strategies are needed to address this complex health problem.

## Background

In this era of epidemic obesity excessive weight gain during pregnancy is of increasing public health concern. It is well known that maternal overweight and obesity is associated with adverse maternal and neonatal outcomes, but the impact of excessive weight gain during pregnancy itself can also have significant health consequences. Excessive maternal weight gain during pregnancy is associated with a number of adverse pregnancy outcomes including increased risk of pre-eclampsia, caesarean section, instrumental delivery, preterm delivery and gestational diabetes[1-5]. There are risks also to the infant of hyperglycaemia, hyperbilirubinaemia and macrosomia[4,6,7]. Excessive weight gain in pregnancy is an important predictor of long-term obesity[8]. Mothers who gain more weight during pregnancy have also been found to have children at higher risk for overweight in early childhood[9].

Over the two decades since the Institute of Medicine (IoM)[10] first issued guidance on healthy weight gain there has been a striking increase in the prevalence of maternal overweight and obesity. Trends in excess weight gain have increased steadily across all population groups. Several studies on gestational weight gain in the USA and Europe indicated that about 20% to 40% of women are gaining weight above the recommendations. A longitudinal survey of 12,583 women in Southampton, UK found that 43% gained excessive weight in pregnancy. This was most common amongst women with a high BMI before pregnancy[11].

Weight management strategies are increasingly focusing on pregnancy as a potentially key time to target weight management to address the rapidly increasing prevalence of obesity in the population. Pregnancy may be a time when behaviours can be challenged with the aim of not only improving the woman's health but also the health of her baby; this being a powerful motivational factor. Interventions have been effective in promoting smoking cessation during pregnancy[12]. There is however a lack of guidance, with regard to a safe and effective approach to the prevention of excessive weight gain in pregnancy, to inform current practice. Targeting diet and exercise behaviours may be effective during this key time. though the relationship between pregnancy, obesity and health risks is not completely clear. There is evidence that factors such as socioeconomic status and ethnicity may be confounding the reported association of excessive weight in pregnancy and poor perinatal outcomes[13,14].

The purpose of this systematic review is to explore the existing quantitative research evidence regarding the effectiveness of dietary interventions with or without physical activity in reducing the risk of excessive weight gain in pregnancy. Perceptions of obesity, food and nutrition are however socially bound. They are viewed differently by different groups of women and the social context in which people live may influence the success of dietary or physical activity interventions for pregnant women[15]. For this reason the review also includes a review of qualitative research to aid understanding of the contextual factors that may influence the effectiveness of interventions.

## Methods

This review adopts an approach incorporating both quantitative and qualitative data previously pioneered by the EPPI centre[16,17]. It includes a review of controlled clinical trials designed to assess the effect of interventions to prevent excessive weight gain during pregnancy. Secondly it draws on qualitative research that explored the views, perceptions and beliefs of health professionals, pregnant women, their partners and families; regarding diet, physical activity and weight management in pregnancy. We included trials conducted in any country, but we drew only on qualitative studies conducted in the UK in order to assess the applicability of interventions to our own context. Finally both analyses were integrated so that findings from the qualitative studies can inform and illuminate the quantitative findings.

### Search

A comprehensive literature review of both published and unpublished 'grey literature' was undertaken to identify relevant studies and background information. Eleven databases were searched and the citation list of relevant review articles and included papers were also searched. The searches were undertaken in early December 2008 and a second search, updating the existing review was conducted in January 2010. Searches were limited by year (1990-2010) corresponding with introduction the concept of excessive gestational weight gain by the IoM (1990).

The search strategy combined terms for pregnancy and terms for body composition, obesity and weight change. This set of "population" terms was then combined with terms for diet, exercise, physical activity advice and monitoring, giving four separate sets of results for each database. Both free text and subject terms were used in the database searches. A sample search strategy for Medline and a list of the databases searched can be found in Appendix 1. In addition a bibliographic search of all the included studies was carried out and experts in the field were also consulted to identify any additional literature.

We included randomised controlled trials (RCTs) published in English. Studies undertaken in non OECD (Organisation for Economic Co-operation and Development) countries were excluded. Participants included women aged eighteen years or over either planning a pregnancy or pregnant and considered normal weight, overweight or obese. Studies were excluded if women had underlying medical complications, were pregnant with twins or if women were underweight. Studies evaluating any dietary intervention with or without additional advice or support for physical activity were included. Studies were included if they reported weight related outcomes, dietary and physical activity outcomes or outcomes related to the pregnancy, birth or the infant. Qualitative studies exploring beliefs and perceptions about diet, physical activity and weight management in pregnancy, conducted in the UK, were included.

The search results were screened by one reviewer and all excluded references were checked by a second reviewer. Where insufficient information was present in the title and abstract to determine eligibility, full papers were retrieved for further consideration. All potentially eligible studies were obtained and re-assessed for inclusion. The inclusion of any studies which were unclear was resolved through discussion.

### Data extraction

Separate data extraction forms were developed for the quantitative and qualitative studies in consultation with clinical experts and each was piloted. Data on study methods, characteristics of participants, interventions and relevant outcomes were independently extracted from included trials by two researchers. Data extraction of the qualitative studies was undertaken somewhat differently, with each subjected to repeated independent readings during which it was appraised and its findings summarized in data extraction form. Consideration was given to the ways in which the methodologies used shaped understandings about the barriers and facilitators affecting healthy weight management in pregnancy. Each study was independently reviewed by two researchers and any differences were resolved by discussion.

### Quality assessment

The internal validity of each included controlled study was assessed using the Cochrane Collaboration's tool for assessing risk of bias[18]. This assesses six key methodological domains; sequence generation, allocation concealment, baseline comparability, intention to treat analysis and loss to follow-up and selective outcome reporting. Blinding of participants and treatment providers was not a factor in the quality assessment as it would not be possible to blind to the treatment category assigned. However blinding of the outcome assessor and analyst would be possible and was assessed.

The methodological quality of the qualitative studies were assessed using the assessment tool in the NICE (National Institute for Health and Clinical Excellence) Methods Manual[19]. This tool included 14 main quality assessment criteria designed to aid judgment on the extent to which study findings were an accurate representation of participant's perspectives and experiences. It drew on a range of qualitative checklists and designed questions exploring the theoretical approach adopted, methods of sampling, rigour in data collection, exploration of the role of the researcher in the review, description of the context, reliability of methods and analysis, richness of data, coherence of findings and consideration of relevant ethical issues. A final assessment sorted studies into one of three categories on the basis of quality: high quality (those meeting 12 or more criteria), medium quality (those meeting nine or ten or more), and low quality (those meeting fewer than nine criteria).

### Data Synthesis

The data synthesis was conducted in three stages according to the framework described by Thomas et al (2004)[16]. Firstly, where possible and if appropriate, the results of eligible controlled studies were statistically synthesized in a meta-analysis to assess the effectiveness of the interventions in the controlled trials. Meta-analysis was undertaken using Cochrane Collaboration Review Manager 5.0 software[20]. The standardised mean difference was used to estimate the pooled mean difference in weight gained between intervention and control groups, using a random effects model.

Statistical heterogeneity between trials was assessed using the chi^2 ^test, its corresponding P-value and the I^2 ^test[18]. Sensitivity analyses were performed excluding poor quality trials. Sub-group analyses were performed grouping trials into pre-specified categories.

Secondly, a thematic synthesis of the findings from the qualitative studies was undertaken. Each study was read and re-read to enable the reviewer to familiarise themselves with the study findings and the methods used. Study findings were coded line by line to characterise the content of each line or sentence. After summarising the findings common themes were identified and supporting quotes drawn from the qualitative studies. The review team then drew out the implications for appropriate interventions suggested by each theme.

Thirdly, a methodological and conceptual matrix was constructed to integrate the findings of the two syntheses. The potential implications of the views of pregnant women, their partners, families, communities and the views of health professionals were presented alongside the content and findings of the soundly evaluated interventions.

## Results

### Description of studies

**Figure 1:**
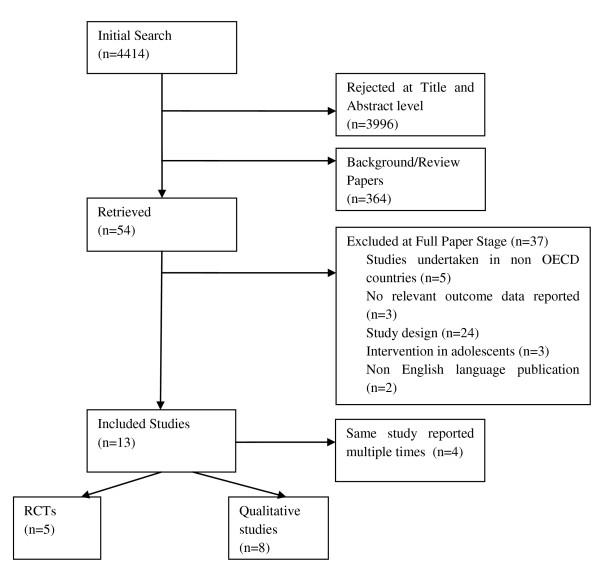
**Flow chart of included studies**.

A total of 13 studies (5 RCTs, 8 qualitative studies) were identified for inclusion in the review. The search yielded 4414 citations. Of these, 3996 were discarded because after reviewing the title and abstract these papers did not meet the inclusion criteria. The full text of the remaining 54 citations was examined in more detail. Thirty-seven studies did not meet the inclusion criteria and were excluded and are described in detail elsewhere[21]. The numbers of papers included and reviewed at each stage were recorded (Figure 1).

### Participants

Five RCT's were included and the number of participants in randomised control trials ranged from 52 to 195 with a total of 577. Baseline characteristics of participants in the RCTs summarised in (table 1). The mean age of the participants ranged from 25.5 to 29 years. Overall mean age of respondents across trials was 27.2 years. The mean pre-pregnancy BMI of participants in the included studies ranged from 22.6 to 34.7 kg/m^2^. Two studies recruited only obese women (BMI ≥ 30 kg/m^2^)[22,23]. Across trials, the number of women included who had previously given birth differed. Where parity was reported, the proportion of primiparous women ranged from 42-47%. Women were enrolled at a mean gestational age that ranged from 9.8 to 15.5 weeks.

**Table 1:** Summary of baseline characteristics

Author, Year	Study Size	Country	Mean Age	First pregnancy%	Mean Pre pregnancy BMI (Kg/m2)	education High school or less%	Ethnicity Non white%	Gestational Age at Enrolment (weeks)
RCTs								

Asbee, 2009[24]	144	USA	26.6	NR	25.5	67%	84%	13.7

Guelinckx, 2010[22]	195	Belgium	29	42%	33.8	NR	0%	9.8

Hui, 2006[26]	52	Canada	26.2	NR	24.5	NR	63.8%	< 26

Polley, 2002[25]	120	USA	25.5	47%	22.6	45%	39%	14.5

Wolff, 2008[23]	66	Denmark	28	NR	34.7	NR	0%	15.5

Socio-economic status or method of assessment was not consistently reported in the studies. Three studies reported educational attainment, the proportion who had only received education up to the end of high school was 67%[24] and 45%[25]. In one trial [26] recruitment occurred only amongst economically deprived populations. The studies also differed in the ethnic profile of their included participants and in their reporting of this data. In Hui et al's study (2006), conducted in Canada, 63.8% of the participants were of aboriginal ethnic origin. In Asbee et al's study (2009), conducted in the USA, 84% were of Hispanic origin. In two European studies all participants were Caucasian[22,23].

Of the 5 included intervention studies, two were conducted in the USA[24,25] one in Canada[26] two in different European countries[22,23] In most studies, women were recruited from obstetric clinics or prenatal services. Wolff et al (2008) recruited through a register of newly diagnosed pregnancies.

### Interventions

The included studies evaluated 'complex interventions' i.e. they contain several interacting components[27]. Most of the interventions included both a dietary and physical activity component. However, Wolff et al (2008) only gave advice about diet. (see table 2 for a summary of interventions)

**Table 2:** Summary of interventions

Study and	Nutrition	Physical Activity	Monitoring weight and behaviour change	Control
Asbee, 2009[24]	▪ 1 meeting with dietician at enrolment: where appropriate food choices discussed and focused food plan given. ▪ Patient focused caloric value divided up as 40% carbohydrate, 30% protein and 30% fat	▪ Instructed to engage in moderate intensity exercise 3-5 times per week	▪ Use of gestational weight gain grid to plot weight at each antenatal appointment. Physician or nurse would inform participant if weight was within IoM guidelines and to modify diet and exercise accordingly.	▪ Routine prenatal care and some educational material containing advice regarding diet and exercise. ▪ Weight measurement at each routine obstetrical appointment.

Guelinckx, 2010[22]	▪ Three, one hour small group sessions led by a nutritionist. Supplemented with purpose designed brochure ▪ Aimed at limiting the intake of energy-dense foods by substituting with healthier alternatives, increasing low-fat dairy products, increasing whole-wheat grains and reducing saturated fatty acids. ▪ Information given about energy balance, body composition and nutrition food labels and techniques of behaviour change to give insight into emotional eating.	▪ Information given on how to increase physical activity	▪ 7 day food diary kept every trimester ▪ Weight measured at each antenatal visit	▪ Routine prenatal care

Hui, 2006[26]	▪ The Food Choice Map (FCM) interview was used as a tool for both assessment and intervention. Participants recalled their usual food intake during 1 week. Dieticians provided a personalized plan for participants, including recommended changes in food choice frequency, portion size and pattern of intake. ▪ FCM software analyzed total energy and macronutrients in daily intake, as well as gestation week-related gain based on information received during the interview.	▪ Instructed in group session exercises and in home based exercise. Groups led by professional trainers and student assistants. Recommended exercise 3-5 times per week for 30 to 45 min per session. Weekly group-based session (~45 min/session). Video exercise instruction was provided to participants to assist with home based exercise.	▪ Information about daily physical activity including a self-recorded activity diary were collected.	▪ Standard care ▪ Physical activity was recommended for participants in the SC group, but they were not instructed in the group exercise sessions or on home-based exercise. ▪ Basic exercise advice that consisted of a simple statement that women should exercise regularly but given no instructions. ▪ Information package about national recommendations for dietary intake during pregnancy

Polley, 2002[25]	▪ Stepped-care behavioural intervention: education and feedback about weight gain, which stressed healthy, low-fat eating Delivered by master's and doctoral level staff with training in nutrition or clinical psychology ▪ Written and oral information in the following areas: (a) appropriate weight gain during pregnancy; (b) exercise during pregnancy (c) healthful eating during pregnancy.	▪ Exercise intervention focused on increasing walking and developing a more active lifestyle.	▪ Newsletters gave advice about exercise as well as diet and sent biweekly. Between visits women were contacted by phone to discuss progress towards the goals set at the previous visit ▪ Personalized graph of their weight gain. Weight changes within the appropriate ranges were informed that they were gaining the expected amount of weight. Weight was measured at every clinic visit and participants advised accordingly.	▪ Usual care/standard nutrition counselling well-balanced dietary intake and advice to take a multivitamin/iron supplement.

Wolff, 2008[23]	▪ Women were instructed to eat a healthy diet according to the official Danish dietary recommendations. ▪ 10 consultations of 1 hour each with a trained dietician during the pregnancy		▪ Seven-day weighed food records were obtained at inclusion, and at 27 and 36 weeks of gestation in both groups. Weights monitored at 27, 36 weeks	▪ The control group had no consultations with the dietician ▪ No restrictions on energy intake or gestational weight gain

In all of the included trials the intervention was delivered by a health care professional with particular expertise in nutrition/psychology or public health. Most commonly this was a dietitian. Wolff et al (2008) offered the most intense contact with 10 consultations of one hour duration with a dietitian. In contrast Asbee et al (2009) offered only one session with a dietitian at enrolment.

Some of the interventions combined face to face dietary counselling with additional supportive material such as newsletters and phone calls[25] supportive software packages[26] and use of food diaries[22,23]. Regular monitoring of weight, use of weight charts and plotting weights for feed back to participants was reported in two studies[24,25].

The nature of the advice given regarding diet appeared to be based on accepted principles of healthy eating including eating at least five portions of fruit and vegetables per day, high fibre bread and limiting intake of high energy snacks of low nutritional value.

One study [26] provided group exercise sessions which participants could join. In three studies, women were advised to develop a more active lifestyle by, for example, increasing walking or engaging in moderate intensity exercise 3-5 times per week[22,24,25]. Methods to record levels of physical activity were used in one study[26].

All of the trials compared the intervention with usual or standard antenatal care. The duration of the intervention was from recruitment in early pregnancy to delivery. One RCT followed up participants four weeks after delivery [23] and another at eight weeks[25].

### Quality of included studies

All the RCTs were described as randomised, although the method of randomisation was confirmed to be adequate in three trials[23-25]. Only one trial[24] used adequate allocation concealment. None of the RCTs described blinding of assessors at outcome evaluations.

Two RCTs [23,25] reported loss to follow-up by treatment arm. Two trials [24,26] reported loss to follow-up but did not report the number lost from each arm. Where this was reported there was not an imbalance in the numbers lost from each group or the reasons for withdrawing from the study. The proportion of participants who were not included in analysis because of withdrawing from the study or being excluded ranged from 8.3% to 34.6% (table 3).

**Table 3:** RCT Quality Assessment

	Adequate sequence generation	Allocation concealment	Blinding at outcome assessment	Incomplete outcome data due to drop-outs during the study or exclusions from the analysis n/N (%)	Baseline comparability
Asbee, 2009[24]	Yes	Yes	No	44/144 (30.6%)	Yes

Guelinckx, 2010[22]	No	No	No	45/130 (34.6%)	Yes

Hui, 2006[26]	Method NR	No	No	7/52 (13.5%)	Yes

Polley, 2002[25]	Yes	No	No	10/120 (8.3%)	Yes

Wolff, 2008[23]	Yes	No	No	16/66 (24.2%)	Yes

NR: not reported

### Gestational Weight Gain

Gestational weight gain was calculated using self reported pre-pregnancy weight and final value weights were based on final weight before delivery or on the day of delivery[22].

**Figure 2:**
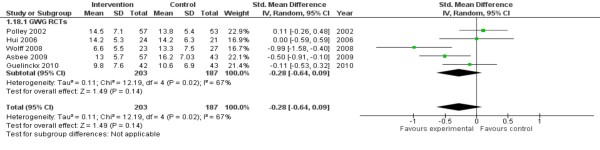
**Gestational Weight Gain - Summary Finding**.

Meta-analyses of 5 RCTs, assessing 390 participants, found no significant evidence that dietary interventions with or without additional support to increase physical activity were effective in reducing gestational weight gain (-0.28 95% CI -0.64 to 0.09). There was substantial and statistically significant heterogeneity present in this analysis (I^2 ^67% p = 0.02) (Figure 2).

### Sub-group and sensitivity analysis

Subgroup analysis was undertaken to explore both heterogeneity and also the impact of factors relating to the context of the study that may influence the effectiveness of the intervention; including pre-pregnancy BMI and socio-economic status of participants.

Subgroup analyses according to baseline BMI status did not demonstrate any difference in the effect of the intervention. A meta-analysis of women with a normal baseline BMI (i.e. 18.5 - 24.9 kg/m^2^) showed no evidence of a difference in the effect of the diet and/physical activity interventions in women with a normal weight at baseline (-0.56 kg 95% CI: -2.84-1.72). There was no evidence of heterogeneity in this meta-analysis. Two studies included only obese women with a BMI ≥ 30 kg/m^2 ^at baseline. When combined in a meta-analysis the two RCTs [22,23] showed no statistically significant difference between intervention and control groups and substantial statistical heterogeneity (I^2 ^= 91%). The effects of different features of the intervention were also explored in a sensitivity analysis including the effects of offering exercise classes and the impact of using regular weight monitoring with feed back to participants. The small number of studies limited the exploration of the effects of different features of the interventions but no evidence was found to indicate which aspects of the interventions may or may not have hindered or enabled the effects of the interventions.

The sensitivity analysis did not demonstrate differences in treatment effects.

The effects of adequate sequence generation in the RCTs were explored. Wolff et al (2008) and Asbee et al (2009) both described using adequate methods for sequence generation and Asbee et al (2009) also described methods for allocation concealment. In a sensitivity analysis these studies showed a statistically significant positive effect in the intervention group with a mean difference in gestational weight gain of -4.71 kg (95% CI -8.11 to -1.91). There was however substantial heterogeneity in this result (I^2^= 58% p = 0.007).

### Qualitative Studies

Nine qualitative papers reporting eight studies were identified and included in this review (table 4)[28-36]. Women expressed many different views and attitudes to diet, physical activity and weight gain in pregnancy. Three themes emerged in the analysis of these studies relating to women's views of weight management in pregnancy; contradictory messages, pregnancy as a time of transition and change and a perceived lack of control.

**Table 4:** Characteristics of Qualitative studies

Study	Aims	Methods	Pregnancy History	Age range	Marital Status	Indicator of se status	Ethnicity
Gross & Bee 2004[29]	To examine the effect of pregnancy on women's recreational activity patterns and to explore pregnant women's beliefs and information sources regarding physical exercise participation	N = 51 Survey and interviews at 16, 25, 34, and 38 weeks gestation lasting 1.5 hours (recorded). Thematic analysis	Previous pregnancies: No previous pregnancy = 40 (70%) Previous miscarriage = 10 (18%)Termination = 7 (12%)	Range 15.7 to 38.2 years (mean 26.3 SD 5.2)	Married = 37 (65%) Cohabiting = 8 (14%) Single = 12 (21%)	Education: Up to 16 years = 32 (55%) Tertiary/professional = 11 (19%)	NR

Fairburn & Welch 1990[30]	To describe the changes in eating habits and attitudes to shape and weight during pregnancy. To determine whether there was a difference with respect to these changes between those women who have previously been concerned about their shape and weight and eating and those who have not.	N = 50 Semi-structured interviews	Primigravida inpatients on post-natal wards; birth within previous 3 days. Mean body mass pre-pregnancy: mean 21.9 (SD = 3.1) 3 had BMI > 25; 3 had BMI < 20 4 had history of bulimia nervosa Mean weight gain = 14.1 kg (SD = 4.1 kg, range 6-25 kg).	Age: mean 25.3 years (SD = 5.3) range 18-37	Married = 42 (84%) Single = 8	Social class: I = 0; II = 24%; IIIa = 52%; IIIb = 12%; IV = 8%; V = 4%	NR

Fox & Yamaguchi1997[31]	To examine the relationship between pre-pregnancy body weight and body image change in primigravid women	N = 76 Anonymous questionnaire and interviews. Thematic analysis	Prepregnancy BMI: Normal weight n = 42; mean 21.55 (range 20-24) Overweight n = 34; mean 29.24 (range 25-39) Weeks gestation: Normal weight n = 42; Mean 35 (range 30-41) Overweight n = 34; Mean 36 (range 30-42) Prepregnancy to current weight gain: Normal weight n = 42; Mean 11.95 (range 9-15) Overweight n = 34; Mean 12.27 (range 10.4-14.5)	Range: 18-27 years	NR	Professional = 6 (8%) Intermediate = 20 (26%) Skilled non-manual = 3 (4%) Skilled manual = 3 (4%) Partly skilled = 1 (1%) Unskilled = 17 (22%) Unemployed or not in paid employment = 12 (16%)	White = 57 (75%) Black = 16 (21%) Indian Asian = 3 (4%)

Johnson et al 2004[35]	To provide more useful insights on the impact of bodily changes during the transition to motherhood (previous research has been contradictory), using IPA.	N = 6 In-depth interviews IPA (Interpretative Phenomenological Analysis)		Ages between 26-34	6 married and living with husbands	4 educated to > degree level	1 British Asian, 5 White

Levy 1999[32]	To map the process involved when women make informed choices during pregnancy	N = 12 Observation and tape recordings of 'booking' interviews between women and midwives. These were transcribed and data considered to be related to decision making was analysed, and also used to trigger conversation in the follow up interviews. Interviews were tape recorded and transcribed verbatim. **Data analysis:** Grounded Theory	Sample: Women attending antenatal clinics in a variety of maternity settings in England 5 primigravada 9 one child 3 two children 3 three children 3 four children	Age range 20 - 38 years.	All women except 1 were in a supportive relationship.	Occupations: Housewives, bank clerk, secretaries, local government officer, farmer, publishing representative.	All British, Caucasian apart from 1 woman of Chinese origin.

Warriner 2000[33]	To examine how the experience of being weighed throughout pregnancy affects women	Interview schedule with prompts. Tape recorded and transcribed; notes made throughout. **Data analysis:** Qualitative content analysis to identify themes and patterns (Polit & Hungler 1995)	Sample: 10 interviewed, 6 in focus group (we are not told whether any of these are the same women). Convenience sample from women attending 2 separate mother and toddler groups (self-selected) No baseline characteristics given	NR	NR	NR	NR

Wiles 1998[36]	To examine the beliefs of women above average weight about appropriate levels of weight gain in pregnancy	**Data Collection:** Interpretative qualitative based on Grounded Theory. **Data analysis:** Transcripts were read several times and coded, then themes were identified which were pursued in subsequent interviews. Further analysis clarified these themes	Sample: 37 Overweight pregnant women of > 30 weeks gestation. Age range = 16-35 years No. of children ranged from 0-3 Weight range 70-138 kg prior to pregnancy (mean 91 kg) Mean prepreg BMI = 32 Weight change at 30 weeks: 2 women lost weight Up to gains of 33 kg None were referred to Dietitians, all were given the same recommendations re weight gain.		30 (81%) lived with partners in independent households. 6 lived with parents 1 lived alone	25 (67%) came from social classes III-V	All white and able-bodied.

Heselhurst et al 2006[40]	To gain a detailed understanding of healthcare professionals' perceptions of the impact that caring for obese pregnant women has on maternity services.	N = 33 Interviews with one (face-to-face) or more member of staff (focus group or discussion meeting). A confirmatory focus group was held to discuss final themes and ensure data saturation. Systematic thematic content analysis (Burnard 1991) adapted from Grounded Theory approach.					

NR: not reported

Three of the studies included were of very good quality[29,31,34] four were of good quality[32,33,35,36] and one[30] was deemed of poor quality mainly because of lack of method detail.

A consistent theme was the contradictory nature of information available to women regarding weight management during pregnancy. Where advice was given it addressed healthy eating rather than weight management issues. Information, when given, was also often contradictory and confusing:

'They recommend swimming and yoga but little else. There's no black and white about what you should and shouldn't do so I don't, I can't follow it at all'. (Gross & Bee 2004, pg 165)

Women reported that information and advice came from three main sources during pregnancy; family and friends, the media and health professionals. Advice about healthy dietary patterns and physical activity behaviours in pregnancy appeared to be strongly influenced by the views of the peer support structures around women during pregnancy. Women reported strong encouragement to rest and to increase their intake of certain food types such as milk and cheese.

Professionals themselves were often embarrassed to initiate a discussion around weight management due to the perceived sensitivities of overweight or obese women. They feared 'victimising' women, and women withdrawing from antenatal care as a consequence.

Pregnancy as transient and a transitional time emerged as a theme from the data. In terms of health behaviour pregnancy is seen as a unique time, when the needs of the unborn child take precedence over the mother's needs, and a time of transition with temporary dietary cravings, nausea and physical discomfort shaping patterns of behaviour. Women expressed ambivalence toward eating behaviour; justifying over-eating during pregnancy as a temporary stage. Some women welcomed the freedom they perceived that pregnancy gave them to eat without limitations, with excess eating being perceived to be positive for the baby.

Women described a general decline in physical activity during pregnancy. A range of factors contributed to this including; anxiety about risks to the unborn baby, general physical discomfort, discouragement to undertake physical tasks by people around them, poor access to exercise facilities and a sense that pregnancy was a time to take it easy and opt out of certain tasks.

Attitudinal changes also occur related to a great extent to pre-pregnancy factors. Women who reported no change in body image perceptions during pregnancy generally had positive body images and a lack of concern with weight prior to conception. These changes are both positive and negative and can change across the duration of the pregnancy. For overweight and obese women pregnancy can be a time where they feel more comfortable with their body image. Pregnancy was seen as a time when being large was socially acceptable and therefore conferred a sense of confidence that had been lacking in their non-pregnant state.

'Before I was pregnant I must have tried every diet possible and people do expect you to diet if you are big. Now I have a wonderful excuse to be big'. (Fox & Yamaguchi 1997, pg 38)

In contrast negative attitudes to body change were mainly reported by women of normal weight who perceived their new pregnant shape as less physically attractive, uncontrollable, attention-provoking and limiting in respect to certain activities. Women used negative language such as 'fat, 'bloated' and 'frumpy' to describe their pregnant state.

A second theme that emerged was the sense of loss of control women experience during pregnancy. This included the more passive role they were sometimes encouraged to take, food provided by women's mothers and women encouraged to rest. Some described weight gain as an inevitable and desirable and not something over which they could exert much control.

'It's just one of those things that you expect happens when you are pregnant, you almost hand your body over to these people and you just accept whatever they say or do to you without really questioning it'. (Warriner 2000 pg. 621)

Women described more restricted access to gym facilities and normal physical activities were less available to them. As well as limitations imposed, the physical demands of pregnancy restricted activity and influenced dietary patterns. Feelings of fullness, nausea or hunger and physical discomfort in later pregnancy all contributed to changing a woman's normal patterns of behaviour.

### Synthesis of quantitative and qualitative findings

The findings of the quantitative and qualitative reviews were juxtaposed to explore the extent to which the interventions responded to the factors identified in the qualitative studies that influence dietary and physical activity health behaviours in pregnancy.

The qualitative studies allowed insight into the experiences of diet and physical activity of women during pregnancy. Women's attitudes and consequent behaviours varied considerably and were influenced by her pre-pregnancy behaviours and attitudes. These were influenced and shaped by her social context. Interventions need to be responsive to the context in which women will be experiencing pregnancy. The RCTs described in this review delivered tailored advice which aimed to incorporate women's preferences, however, the effects were either so small there is at present insufficient numbers of studies to demonstrate an effect, or they have no effect. The evidence from this review suggests that targeting women in pregnancy is not likely to be sufficient to make a substantial difference to reducing excessive weight gain in pregnancy.

Some aspects of the interventions did address issues raised by the qualitative studies. The lack of information or contradictory information was addressed by all of the interventions. Giving consistent information throughout pregnancy and delivering it in a variety of formats did not make a substantial difference in the included studies. Women's health behaviours were influenced by the beliefs and attitudes of her partner, peers and wider family. This exerts a powerful influence and may serve to undermine the messages of health professionals. Interventions at a community level may support interventions that are targeting the behaviour of individuals.

None of the interventions trained those professionals involved in delivering antenatal care with specific skills to address issues of healthy dietary and physical activity behaviours in pregnancy. Instead they relied on nutritionists, dieticians or fitness instructors to deliver the interventions. It was clear that health professionals themselves felt uncomfortable addressing issues of weight management in pregnancy particularly with women who were overweight or obese. The health messages therefore may not have been consistent.

The interventions in the included trials did not seek to address the wider, social factors that contribute to poor weight management, such as ready access to energy dense foods, increasing reliance on cars, a shift towards physical inactivity and unequal exposure to health damaging aspects of our environment. Pregnancy did appear to be a time of change, when women were adopting behaviours that were perceived to be better for the baby. Many women also described it as a time when they sensed a loss of control and a time of transition, after which normal patterns of dietary limitation and exercise would resume. The dietary cravings and physical limitations experienced by some women may also increase a sense of powerlessness. Facilitating behaviour change may be more effective amongst women where a sense of control is felt and interventions delivered in such a way as to re-establish a sense of control.

The interventions all assumed compliance with the underlying values implicit within them - i.e. that weight gain and overweight is not good. For some women these may be attitudes that are hard to accept, pregnancy may be a time when they feel comfortable, able to eat with fewer limitations and overweight being more socially acceptable. As such health messages may not have been accepted and adopted by participants.

## Discussion

The primary meta-analysis of 5 RCTs found that interventions to prevent excessive weight gain during pregnancy showed no clear evidence of effect or lack of effect. There was substantial heterogeneity (I^2 ^= 67%) in this analysis. The interventions comprised strategies to promote both dietary patterns and physical activity behaviours that would assist in preventing excessive gestational weight gain. In most instances these combined a range of tailored and intense interventions with information delivered in accessible ways to women. In one study only dietary changes were implemented[23].

Additional subgroup and sensitivity analysis did not find that women's pre-pregnancy weight, or features of the intervention or study design appeared to influence the effectiveness of the interventions.

The synthesis of both the quantitative and qualitative evidence reveals what may help or hinder the success of the interventions in preventing excessive weight gain in pregnancy. Gaps include a lack of interventions which seek to educate and inform the wider family and social network surrounding pregnant women. Lay beliefs about nurturing behaviours during pregnancy to benefit the baby may contradict messages from health professionals. Interventions also need to seek to train and prepare health professionals to counsel women about healthy weight gain in pregnancy. Pregnancy is a time of considerable change, and while it is a time when women alter behaviours for the benefit of the baby, it also appears to be a time when messages about preventing excessive weight gain are less welcome. For some overweight women this is a time where they enjoy greater self confidence. The evidence of the effects of the interventions is still limited to a small number of studies and further research is needed in order to explore what types of interventions are effective in what contexts and for which women.

### Comparison with other studies

Four systematic reviews on the effects of dietary and lifestyle interventions in pregnant women have been published. Two reviews identified only two trials of interventions during pregnancy and were unable to draw conclusions about the effectiveness of interventions due to the lack of evidence[37,38]. Two recent systematic reviews included both non-randomised and randomised controlled studies[21,39]. Streuling et al (2010) combined the effects of non-randomised and randomised studies concluding the interventions demonstrated a statistically beneficial effect (standardized mean difference of -0.22 units (95% CI: -0.38, -0.05 units). The analysis of randomised controlled trials did not show a statistically significant effect. Campbell et al (2010) concluded that the heterogeneity between studies, and the methodological weaknesses of the included non-randomised studies did not support pooling the data.

### Strengths and limitations of the study

The strengths of this review include the comprehensiveness of the searches, the rigorous synthesis methods used and the inclusion of data from qualitative research along-side controlled trials which allowed us to not only explore effectiveness but also the factors that may help or hinder effectiveness. The small numbers of studies are a limitation of the available body of research, as is the lack of intervention studies conducted in the UK. The qualitative data conducted in the UK was juxtaposed alongside trial data which was not carried out in the UK. Assuming that the qualitative data illuminates findings of work conducted in culturally different settings is a limitation of this approach.

### Implications for further research and clinical practice

Our findings suggest that behaviour change interventions may be more effective if there are also efforts to target communities and seek to change social attitudes to diet and exercise in pregnancy. Health messages need to be clear and consistent and research evaluating action at a community level should be developed.

There is also a need for UK based intervention studies that are evaluated using robust methods that are well reported. Methods to address blinding of outcome assessment and ensure allocation concealment are particularly necessary. Cluster randomised trials may be appropriate for this type of intervention. Trials also need to have larger sample sizes from representative populations. The included studies showed no evidence of effect for gestational weight gain suggesting it would be valuable to explore potential barriers to effective interventions using qualitative research methods so that more effective interventions can be designed. Trials are also needed with adequate follow-up to assess the impact on weight retention post partum at 6, 12 and 18 months.

In view of the poor methodological quality of the included studies, it is difficult to draw any definite conclusions about the efficacy or lack of efficacy of diet and physical activity interventions for pregnant women and therefore guide clinical practice and policy making. There is no robust evidence that supports or rejects the theoretical view that pregnancy is a 'teachable moment' in preventing excessive weight gain. There is also no evidence to suggest there are any adverse effects as a result of the interventions.

## Conclusion

There is a lack of sufficient evidence to conclude that interventions are effective in reducing gestational weight gain. There is also no evidence to suggest there are any adverse effects as a result of the interventions. The lack of effect may reflect the failure of the interventions to address some of the barriers to healthy weight gain identified in the qualitative studies. Future interventions that challenge lay beliefs about health behaviours in pregnancy and strategies that enable women to maintain physical activity during pregnancy should be developed and evaulated. Strategies should also engage health professionals working with pregnant women so that messages are consistent and professionals are equipped with the necessary skills to address weight management in pregnancy. Additionally research should explore further the potential for greater effectiveness of interventions amongst obese women, the long term outcomes of interventions and the value of pre-pregnancy interventions.

## Competing interest Statement

All authors have completed the Unified Competing Interest and declare: all authors had financial support from the National Institute for Health and Clinical Excellence (NICE) for the submitted work; no financial relationships with any other organisations that might have an interest in the submitted work in the previous 3 years; no other relationships or activities that could appear to have influenced the submitted work.

## Authors' contributions

FC, MJ and JM identified the studies for inclusion in the review and extracted data. FC and MJ conducted the analysis and FC drafted the review. LG designed and undertook the literature searches. LG participated in the design and coordination of the review. All authors participated in proof reading the review.

## Pre-publication history

The pre-publication history for this paper can be accessed here:

http://www.biomedcentral.com/1471-2458/11/491/prepub
